# Clinical and Genetic Findings in an Autosomal Dominant Optic Atrophy-Compatible Phenotype Harboring an OPA1 Variant: A Case Report

**DOI:** 10.7759/cureus.95622

**Published:** 2025-10-28

**Authors:** Ricardo A Murati Calderon, Gabriella Landestoy, Natalio Izquierdo

**Affiliations:** 1 Department of Ophthalmology, School of Medicine, University of Puerto Rico - Medical Sciences Campus, San Juan, PRI; 2 Department of Surgery, School of Medicine, University of Puerto Rico - Medical Sciences Campus, San Juan, PRI

**Keywords:** autosomal dominant optic atrophy, inherited optic neuropathy, opa1 gene, spectral domain optical coherence tomography, variant of uncertain significance (vus)

## Abstract

We report a case of an 18-year-old Hispanic male patient with clinical features consistent with autosomal dominant optic atrophy (ADOA), including bilateral optic disc pallor, childhood color deficits, and visual field loss. The patient reported one year of progressive blurry vision; best-corrected visual acuity was measured at 20/30 in the right eye (OD) and 20/60 in the left eye (OS). Multimodal imaging revealed the expected structure-function pattern, with spectral-domain OCT demonstrating predominant retinal nerve fiber layer (RNFL) thinning and a preserved macular contour. Meanwhile, Humphrey's visual fields showed paracentral defects in the OD and a superior arcuate defect with inferior scotomas in the OS. Genetic testing identified a heterozygous *OPA1* variant (c.1310A>G; p.Gln437Arg), currently classified as a variant of uncertain significance (VUS). To our knowledge, this variant has not been previously reported from Caribbean cohorts with ADOA. The case underscores the importance of clinical-genetic correlation in interpreting uncertain variants and highlights how limited regional allele-frequency data can constrain classification. Therefore, our case expands the phenotypic and geographic context for *OPA1*-associated optic neuropathy and motivates segregation testing, broader genetic screening, and functional studies to clarify pathogenicity and improve diagnostic accuracy in underrepresented populations such as those in the Caribbean.

## Introduction

Autosomal dominant optic atrophy (ADOA) is one of the most common inherited optic neuropathies [1]. Wolfgang Jaeger first described it in 1954 after observing a German family with clinical signs of optic atrophy that followed an autosomal dominant inheritance pattern [2]. Epidemiological data estimate that the ADOA worldwide prevalence is three per 100,000 in most populations [3,4]. Reported prevalence varies by region, with population studies estimating it at approximately one in 35,000 in the North of England and as high as one in 10,000 in Denmark [3,4]. However, there is a notable lack of epidemiological data regarding prevalence or incidence for Caribbean populations, highlighting a regional data gap that may obscure population-specific or founder variants. Additionally, the lack of Caribbean-specific epidemiologic and allele-frequency data limits variant interpretation and counseling in this region.

Clinically, the disease typically manifests during the first decade of life as slowly progressive central visual loss. Because the disease exhibits variable penetrance, visual acuity can range widely from mild-to-severe vision loss, such as legal blindness [5]. This loss is often accompanied by color vision deficits and central or cecocentral scotomas [6]. On detailed ophthalmoscopic examination, optic disc pallor or atrophy is common; this reflects the selective degeneration of retinal ganglion cells (RGCs) and their axons, a hallmark of the disease [3,6]. Multimodal imaging, such as optic nerve optical coherence tomography (OCT), is key, as it helps demonstrate retinal nerve fiber layer (RNFL) thinning [7]. Furthermore, about 20% of cases report extraocular manifestations, with sensorineural hearing loss, ataxia, and other neuromuscular symptoms being the most commonly reported [8].

Several pathogenic mutations in the optic atrophy 1 (*OPA1*) gene have been described as the cause of ADOA [1]. The *OPA1* gene is a nuclear gene that encodes a GTPase targeted to the mitochondrial inner membrane and intermembrane space. OPA1 is essential for mitochondrial inner membrane fusion and the maintenance of mitochondrial DNA integrity [9]. Pathogenic variants disrupt mitochondrial fusion and cristae structure, leading to mitochondrial fragmentation, impaired oxidative phosphorylation, and an increase in reactive oxygen species, ultimately affecting the RGCs, which are highly vulnerable due to their high metabolic demand [10]. This effect in the RGCs leads to selective degeneration with consequent optic nerve atrophy [10].

We report on a young Puerto Rican male patient with a heterozygous *OPA1* variant (c.1310A>G; p.Gln437Arg), currently classified as a variant of uncertain significance (VUS), who presented with clinical findings of early-onset vision loss and bilateral optic nerve atrophy consistent with ADOA. Furthermore, to our knowledge, this is the first reported case with this mutation in the *OPA1* gene with a phenotype compatible with ADOA in the Caribbean.

## Case presentation

An 18-year-old Hispanic male patient with a medical history of asthma was referred to our clinic by a peripheral ophthalmologist for a genetic evaluation due to bilateral optic nerve atrophy with no attributed etiology after multiple evaluations. The patient has had progressive blurry vision for one year. The patient denied experiencing ocular complaints such as flashes, photophobia, or nyctalopia; however, he reports an ocular history of mild red-green color deficiency since early childhood. There was no family history of eye disease, in particular, no history of optic nerve anomalies. Review of systems, toxic habits, and social history was also unremarkable.

Upon comprehensive ophthalmic evaluation, the patient’s best corrected visual acuity was 20/30 in the right eye (OD) and 20/60 in the left eye (OS). Refraction was -0.75 -0.25 x 150˚, OD, and -0.50 -0.50 x 30˚, OS. Intraocular pressures measured by applanation tonometry were 17 mmHg in both eyes (OU). His pupils were equal, round, and reactive to light, with a noticeable relative afferent pupillary defect OS. Extraocular movements were full in all directions of gaze OU, and without pain. The Color Vision Hardy-Rand-Rittler test revealed a mild red-green deficiency, and confrontation visual fields were abnormal for finger counting in the temporal quadrants of both eyes.

**Figure 1:**
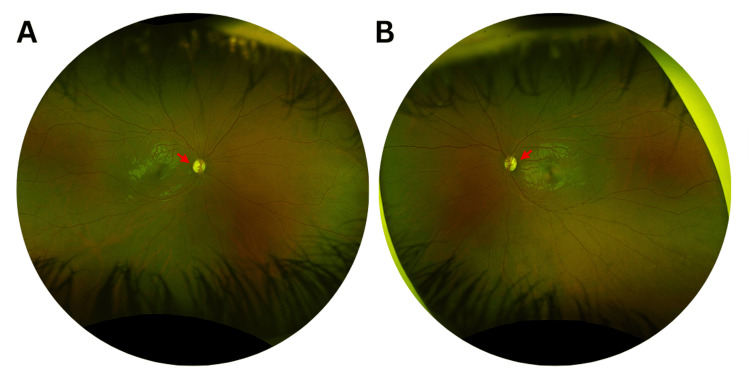
Ultra-widefield fundus imaging of the right and left eyes at presentation Color fundus photographs at initial presentation of the right (A) and left (B) eyes reveal bilateral mild temporal optic nerve pallor (red arrows) and increased optic disc cupping.

The anterior segment examination was unremarkable. Subsequently, a dilated fundus examination (Figures 1A-1B) showed increased disc cupping and bilateral temporal optic nerve pallor in both fundus photographs of the patient.

**Figure 2:**
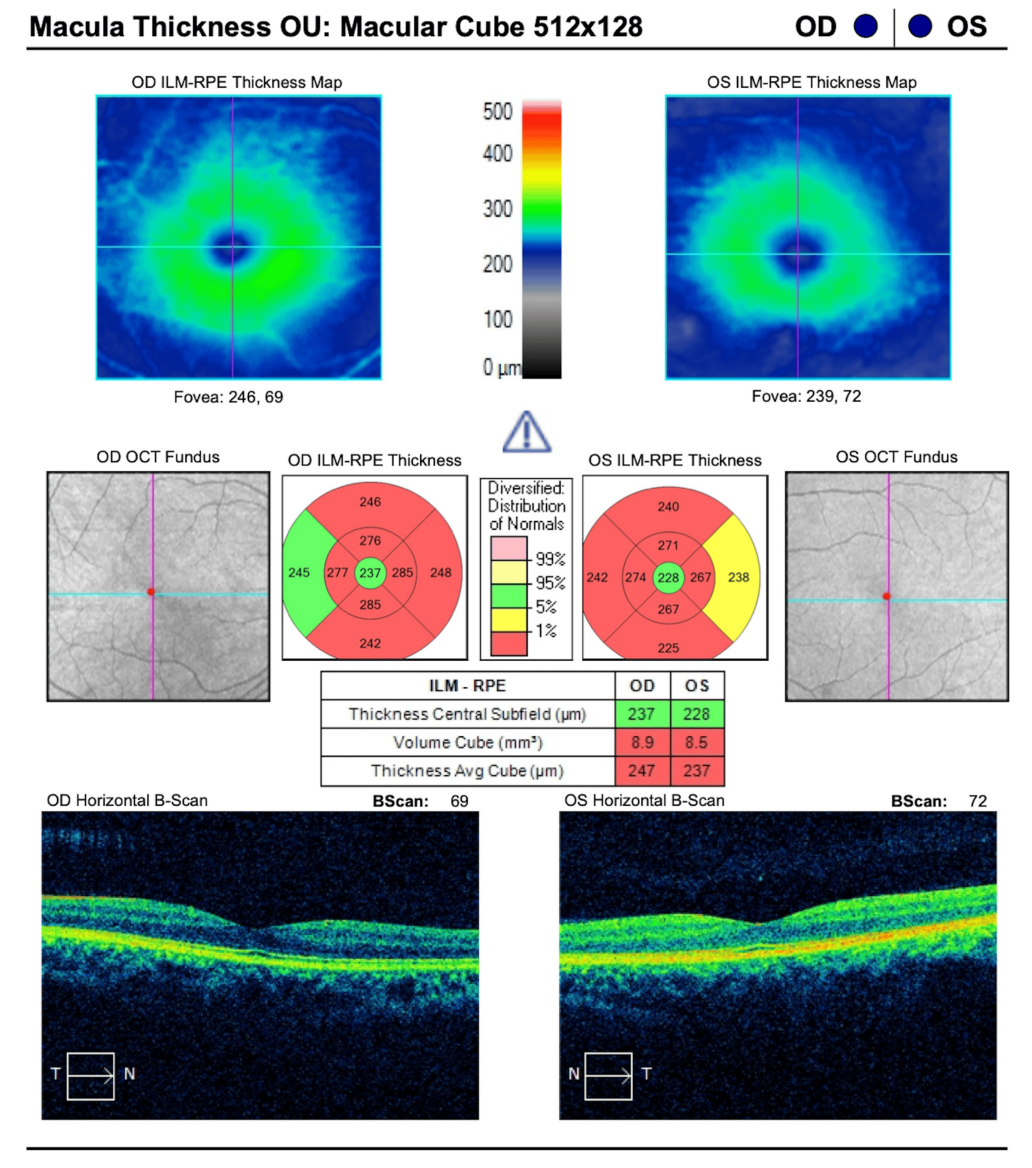
Macular spectral-domain OCT study showing a preserved macular contour with decreased macular thickness and volume in both eyes Conventions: The normative color scale indicates values within normal limits (green: 5th-95th percentile), borderline (yellow: 1st-5th percentile), and outside normal limits (red: <1st percentile) compared to the device's age-matched database. Abbreviations: OCT, optical coherence tomography; ILM, inner limiting membrane; RPE, retinal pigment epithelium

To further investigate structural abnormalities, spectral-domain OCT of the macula (Cirrus; Carl Zeiss Meditec AG, Dublin, CA) (Figure 2) showed a preserved macular contour in both eyes, with a central subfield thickness (CST) of 237 µm and 228 µm in the right and left eyes, respectively. In contrast, cubic volume was slightly decreased in both eyes, with values of 8.9 mm³ and 8.5 mm³ in the right and left eyes, respectively.

**Figure 3:**
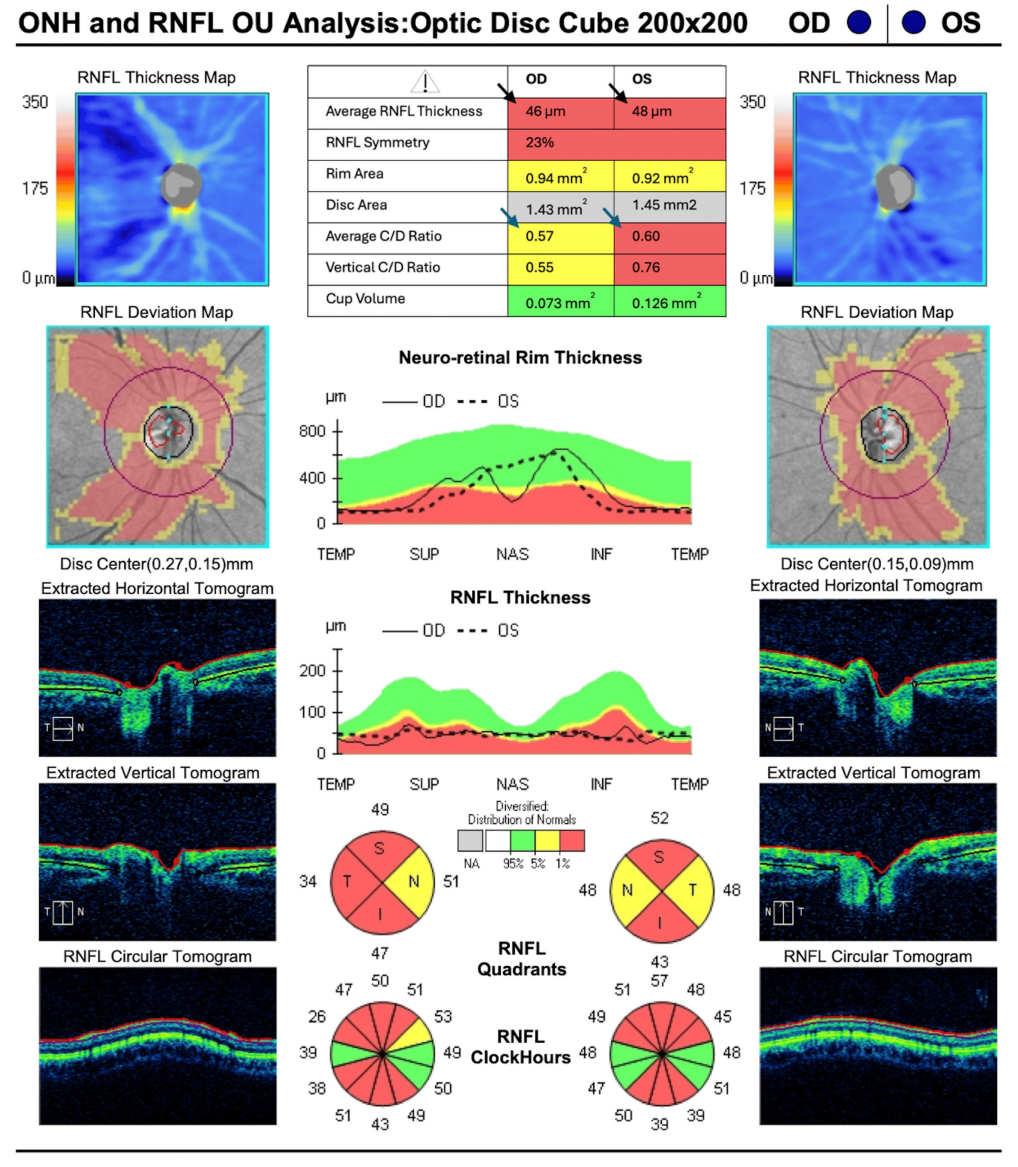
Spectral-domain OCT of the optic nerve Optic nerve OCT demonstrates severely decreased retinal nerve fiber layer (RNFL) thickness (black arrows) with increased cup-to-disc ratio (blue arrows) in both eyes. Conventions: Probability maps utilize the device's age-matched normative database, where green indicates values within normal limits (5th-95th percentile), yellow indicates values borderline (1st-5th percentile), and red indicates values outside normal limits (<1st percentile). Black arrows indicate RNFL sectors below the 1st percentile, predominantly superior, inferior, and temporal; blue arrows indicate enlarged cup-to-disc ratio. Key measurements: Average RNFL thickness 46 μm OD and 48 μm OS (severely reduced vs age-matched norms ~ 80-100 μm); average cup-to-disc ratio 0.57 OD and 0.60 OS. Table keys: Row 1, average retinal nerve fiber layer thickness; Row 2, retinal nerve fiber layer symmetry; Row 3, rim area; Row 4, disc area; Row 5, average cup-to-disc ratio; Row 6, vertical cup-to-disc ratio; Row 7, cup volume Abbreviations: ONH, optic nerve head; OCT, optical coherence tomography; RNFL, retinal nerve fiber layer; C/D Ratio, cup-to-disc ratio; Temp, temporal; Sup, superior; Nas, nasal; Inf, inferior; OD, right eye; OS, left eye; OU, both eyes

Additionally, spectral-domain OCT of the optic nerve (Cirrus; Carl Zeiss Meditec AG, Dublin, CA) (Figure 3) showed a decreased retinal nerve fiber layer (RNFL) of 46 µm and 48 µm in the right and left eyes, respectively. The optic nerve average cup-to-disc ratio was 0.57 and 0.60, in the right and left eyes, respectively.

**Figure 4:**
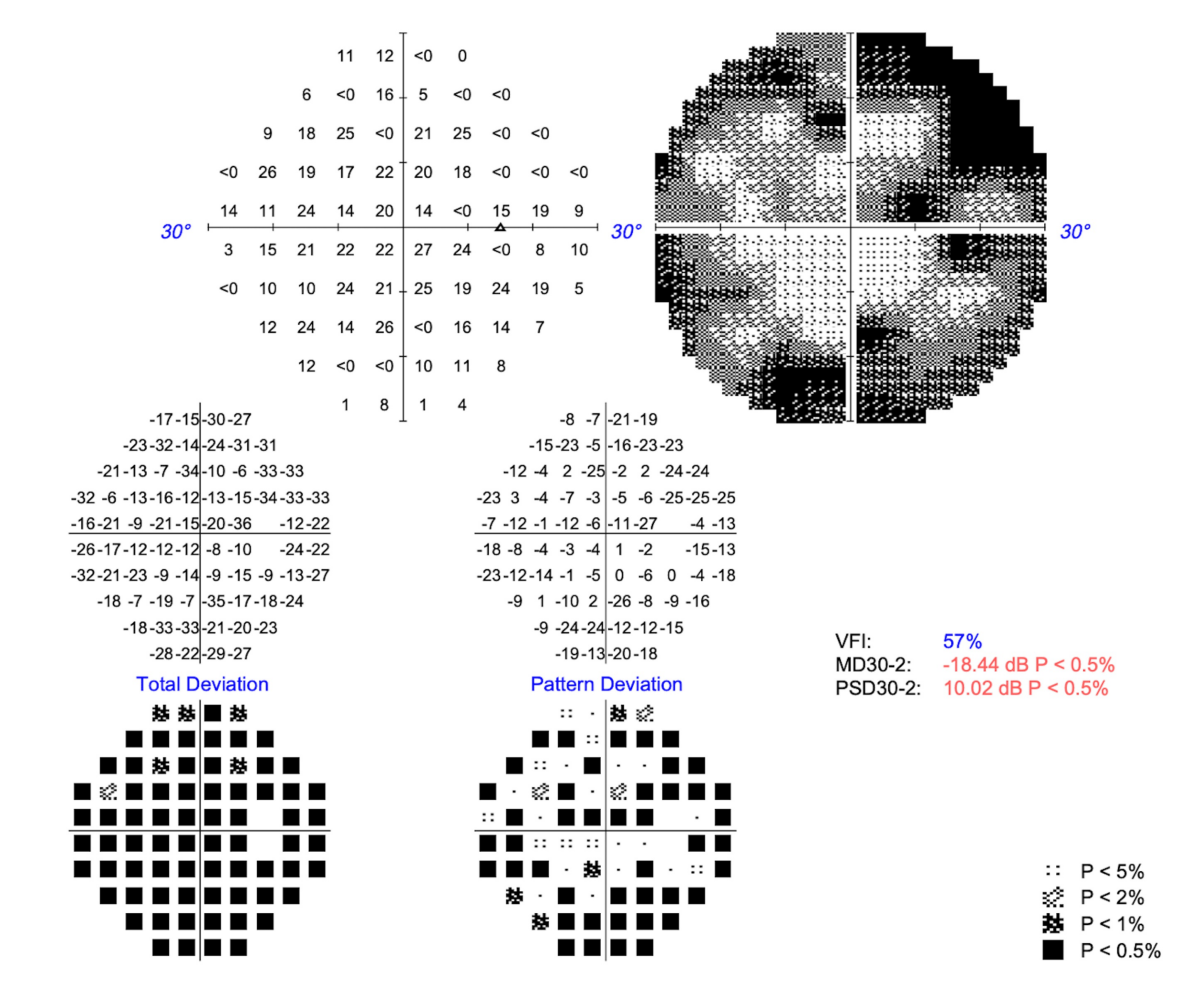
Visual field testing (30-2) of the right eye shows a significantly decreased MD of -18.44 dB (p<0.5%) and a PSD of 10.02 dB (p<0.5%) with a VFI of 57% VFI, visual field index; MD, mean deviation; PSD, pattern standard deviation

**Figure 5:**
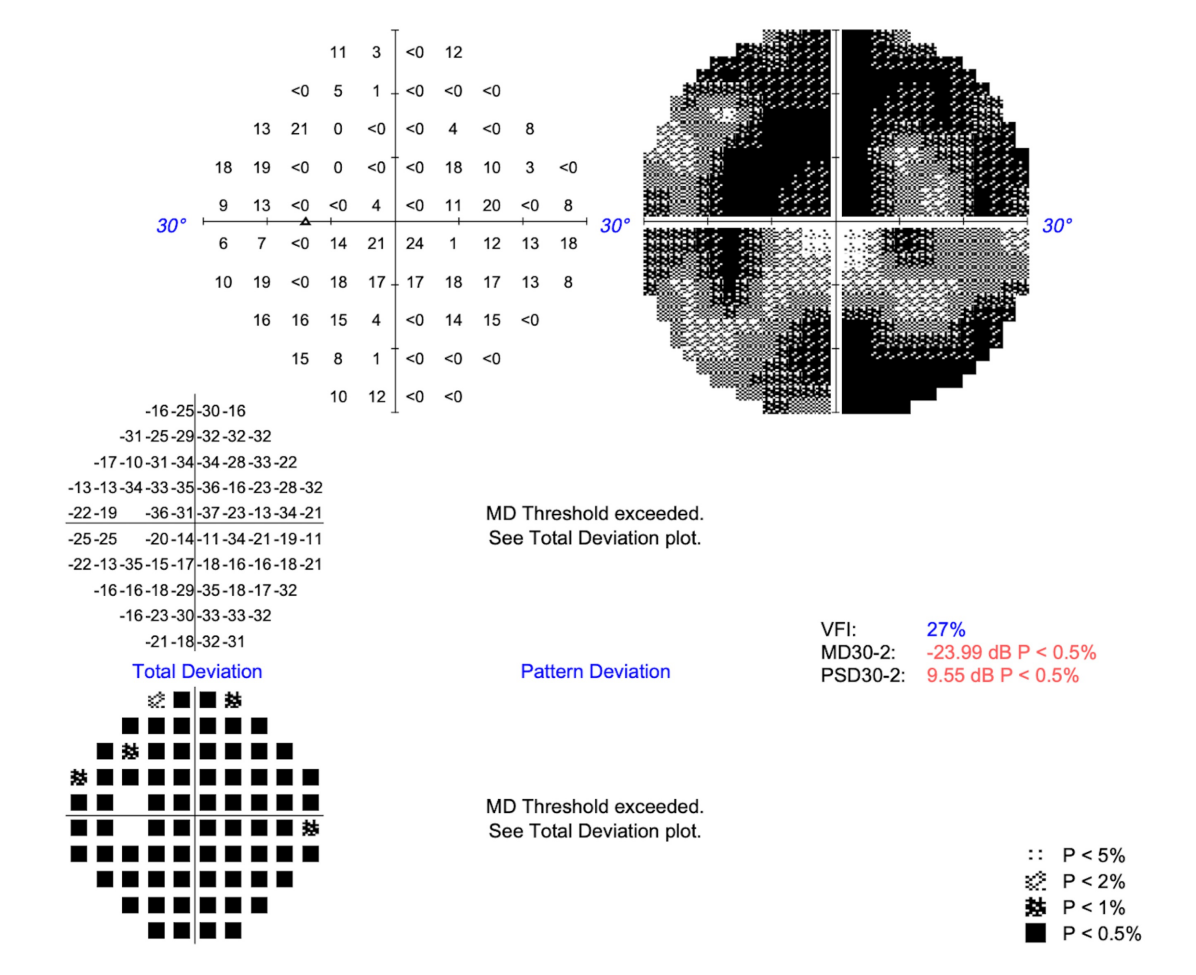
Visual field testing (30-2) of the left eye shows a significantly decreased MD of -23.99 dB (p<0.5%) and a PSD of 9.55 dB (p<0.5%) with a VFI of 27% VFI, visual field index; MD, mean deviation; PSD, pattern standard deviation

Upon Humphrey Swedish Interactive Thresholding Algorithm (SITA) Standard visual field analyses (Carl Zeiss Meditec AG, Dublin, CA), the patient had paracentral scotomas in the right eye (Figure 4) and a superior arcuate pattern and inferior scotoma defects in the left eye (Figure 5). Both had statistically significant mean deviation values of -18.44 dB in the OD and -23.99 dB in the OS (p<0.5%).

Upon the patient’s mother’s ophthalmic examination, she had large optic nerve heads with temporal pallor; however, her visual acuity was adequate and stable with no visual complaints. Based on the characteristic ophthalmoscopic and multimodal imaging findings and findings in his mother, a clinical diagnosis of bilateral optic nerve atrophy secondary to a possible autosomal dominant optic atrophy was made. Following the diagnosis, the patient was counseled regarding the nature of the disease, its possible genetic association with specific genes, and its progressive course. A supportive management approach was discussed, focused on residual visual function. We initiated supportive management, including a daily vitamin B complex regimen, while the etiologic work-up was completed. The patient was referred for genetic counseling to discuss the implications of the autosomal dominant inheritance pattern and to guide future family screening. Despite outreach, the mother later reported that no relatives completed genetic testing; therefore, segregation analysis could not be performed.

Genetic testing was conducted using a saliva sample, and a next-generation sequencing (NGS) diagnostic test (Invitae Inherited Retinal Disorders Panel; Invitae Corporation, San Francisco, CA) was done to evaluate over 330 genes associated with inherited retinal genetic diseases. NGS and deletion and duplication analysis, performed using the Invitae Panel, revealed a heterozygous mutation in the *OPA1* gene. The variant was c.1310A>G (p.Gln437Arg), currently classified in ClinVar as a variant of uncertain significance with conflicting classifications of pathogenicity. According to the gnomAD database, the variant is absent or ultra-rare with no reported frequency. Without segregation or functional data, the classification appropriately remains of uncertain significance.

## Discussion

A meta-analysis involving over 400 individuals with *OPA1 *mutations reported the classic ADOA phenotype, including early bilateral central vision loss, optic disc pallor, and color vision deficits [8]. Ham et al. also identified a syndromic form, "ADOA-plus", in which patients exhibited additional features beyond the classic phenotype, most commonly sensorineural hearing loss and cerebellar/coordination difficulties, and less frequently heart or muscle involvement [8]. Notably, these extra-ocular features occurred more frequently with maternally inherited variants, demonstrating variability in disease presentations [8]. In comparison, our patient's findings align with the classic ADOA presentation rather than ADOA-plus, showing bilateral vision loss during early adolescence, color vision deficiency, and optic disc pallor, but lacking extraocular symptoms. Furthermore, unlike the subgroup with extraocular involvement, neither our patient nor his family reported related symptoms. However, the mother’s exam did reveal large optic nerves with temporal pallor and stable vision, despite no complaints. Thus, while our patient’s presentation matches the typical features described in the literature, his mother’s findings may further illustrate the intra-familial variability seen in ADOA, as she exhibited stable vision despite abnormal exam findings.

Retinal ganglion cells are exceptionally demanding in terms of energy requirements, as they are critically dependent on efficient mitochondrial oxidative phosphorylation [11]. The *OPA1* gene product, a dynamin-related GTPase, is crucial for maintaining mitochondrial integrity; consequently, OPA1-mediated mitochondrial dynamics are vital for the survival of RGCs, axonal integrity, and the maintenance of the papillomacular bundle [6,12]. Loss-of-function *OPA1* mutations disrupt the inner-membrane GTPase activity, impairing mitochondrial fusion and cristae structure, ultimately leading to poor energy production and increased reactive oxygen species in RGCs [9,10]. Clinically, this cascade leads to optic disc pallor, color vision deficits, and early RNFL thinning, explaining the central, cecocentral, and paracentral scotomas typical of ADOA [7]. Thus, the adolescent onset of visual loss, dyschromatopsia, optic nerve pallor, and RNFL loss in this patient matches the underlying biological mechanism, supporting the ADOA diagnosis described in the previous literature.

Building on this mechanistic understanding, ancillary testing in this case mirrors the expected pattern of structural and functional changes in OPA1-related disease. Spectral-domain OCT of the optic nerve demonstrates severe RNFL thinning, with an average thickness of 46 µm OD and 48 µm OS, findings concordant with cohort data linking RNFL loss to visual acuity decrements [7]. Similarly, Humphrey's visual fields reveal central, cecocentral, and paracentral sensitivity loss with superimposed arcuate involvement. This pattern reflects the papillomacular bundle-predominant RNFL loss observed on OCT, rather than glaucomatous defects, and aligns with findings from studies involving pediatric patients diagnosed with ADOA based on *OPA1* mutations [13]. Taken together, the OCT and visual field findings provide structure-function concordance that strongly supports a clinical ADOA phenotype in this patient. The mean deviation values of -18.44 dB OD and -23.99 dB OS indicate severe visual field loss, aligning with values reported in advanced ADOA cohorts [7,13]. The strong concordance between structural RNFL thinning (46 and 48 µm, respectively) and these functional deficits reinforces the characteristic structure-function pattern of ADOA. This pattern distinguishes it from glaucomatous optic neuropathies, which often exhibit arcuate defect loss without corresponding involvement of the papillomacular bundle. Additionally, the preserved macular contour, with only slight macular volume reduction, argues against a primary maculopathy and supports an optic neuropathy etiology.

Despite these supportive clinical, structural, and functional findings, the current genetic data, which identify a VUS, do not establish a definitive molecular diagnosis, indicating that further research or reclassification may be warranted. Thus, while the clinical presentation is clear, the genetic basis in this case remains uncertain.

Genotype-phenotype correlations deepen our understanding of the clinical spectrum associated with *OPA1* variants. Data further suggest that missense substitutions within or near *OPA1’s* critical functional domains, particularly the GTPase region, are enriched among pathogenic ADOA variants and may act via dominant-negative effects - incorporating into normal oligomers but impairing complex activity [1,9]. The heterozygous c.1310A>G (p.Gln437Arg) variant identified in our patient resides in a region where neighboring missense variants have been associated with classical ADOA presentation, including those with broader manifestations, such as sensorineural hearing loss, underscoring the functional importance of this locus. For instance, a report by Leruez et al. described two novel *OPA1* mutations, including a missense substitution at the same codon (p.Arg437Glu), in OPA1-related optic atrophy with hearing loss [14]. Taken together, these findings highlight positional sensitivity around residue 437 and support our case’s rationale for the p.Gln437Arg classification, thus linking the genetic context to our patient’s unresolved diagnostic status.

This report presents the clinical and genetic profile of a patient exhibiting features highly suggestive of ADOA, with a heterozygous VUS, c.1310A>G (p.Gln437Arg) identified in *OPA1*. The patient's clinical presentation - including adolescent onset, bilateral vision loss, color vision deficit, optic disc pallor, significant RNFL thinning, and visual field defects - is consistent with the spectrum of findings previously described in ADOA cases associated with pathogenic *OPA1* mutations. Notably, this specific variant has not been reported in the gnomAD or ClinVar databases, in contrast to other well-characterized pathogenic mutations. In summary, while the clinical phenotype aligns with ADOA, the genetic results remain inconclusive due to the VUS, underscoring the need for further genetic investigation and clarification.

To our knowledge, this is the first reported case of a Caribbean patient with ADOA and this specific *OPA1* variant, highlighting a potential expansion in both the genotypic and geographic spectrum of the disease. These findings raise the possibility that, while its clinical significance remains uncertain, p.Gln437Arg may in fact represent a disease-associated variant, warranting further genetic and functional evaluation.

## Conclusions

To our knowledge, this is the first detailed clinico-genetic description of ADOA in a Caribbean patient carrying the *OPA1* variant c.1310A>G (p.Gln437Arg). Although this variant is currently classified as a VUS, the patient’s phenotype, including painless bilateral visual decline in adolescence, optic disc pallor, RNFL thinning on OCT, and visual field losses, closely mirrors the classic features described in OPA1-related disease. These findings raise the possibility that this variant may be disease-relevant, particularly in light of its locations within or adjacent to *OPA1’s* functional GTPase region and the absence of alternative etiologies.

Our findings underscore the importance of careful clinico-genetic correlation when interpreting uncertain variants in inherited optic neuropathies. Segregation testing, including formal testing of additional relatives, data deposition in public databases, and functional assays, is warranted to clarify pathogenicity. Therefore, this case supports the integration of detailed clinical assessment with genetic analysis to refine the interpretation of variants. Broader genetic screening and the development of Caribbean-specific allele-frequency resources are also necessary to enhance diagnostic accuracy in underrepresented populations.
